# Insanity Amongst the Negroes

**Published:** 1889-03

**Authors:** 


INSANITY AMONGST THE NEGROES.—A crazy negro in the South was scarcely ever heard of in the days of slavery, but now there are many to be found, and the cases are increasing in number. What is the inference, it is that African slavery as it existed in the South was not the cruel and inhuman system that it was represented to be. Now that the negro is free no one desires to re-enslave him, and yet the truth remains that the negro population in the South in the days of slavery were the best cared for and the happiest class of laboring people known to the history of the world. More than this can be trutufully said—the type of civilization as illustrated among the slave holding people of Middle Georgia, previous to the late war, was of a higher order than that of any portion of the North or any other country in the world.

